# Thermocatalytic Pyrolysis of Waste Areca Nut into Renewable Fuel and Value-Added Chemicals

**DOI:** 10.1021/acsomega.3c10184

**Published:** 2024-06-10

**Authors:** Ranjeet Kumar Mishra, Bhavana Gariya, Priyanka Savvasere, Devanshu Dhir, Pradeep Kumar, Kaustubha Mohanty

**Affiliations:** † Department of Chemical Engineering, Manipal Institute of Technology, Manipal Academy of Higher Education, Manipal, Karnataka 576104, India; ‡ Department of Chemical Engineering, Ramaiah Institute of Technology, Bangalore, Karnataka 560054, India; § Department of Chemical Engineering and Technology, Indian Institute of Technology (BHU), Varanasi 221005, India; ∥ Department of Chemical Engineering, Indian Institute of Technology, Guwahati, Assam 781039, India

## Abstract

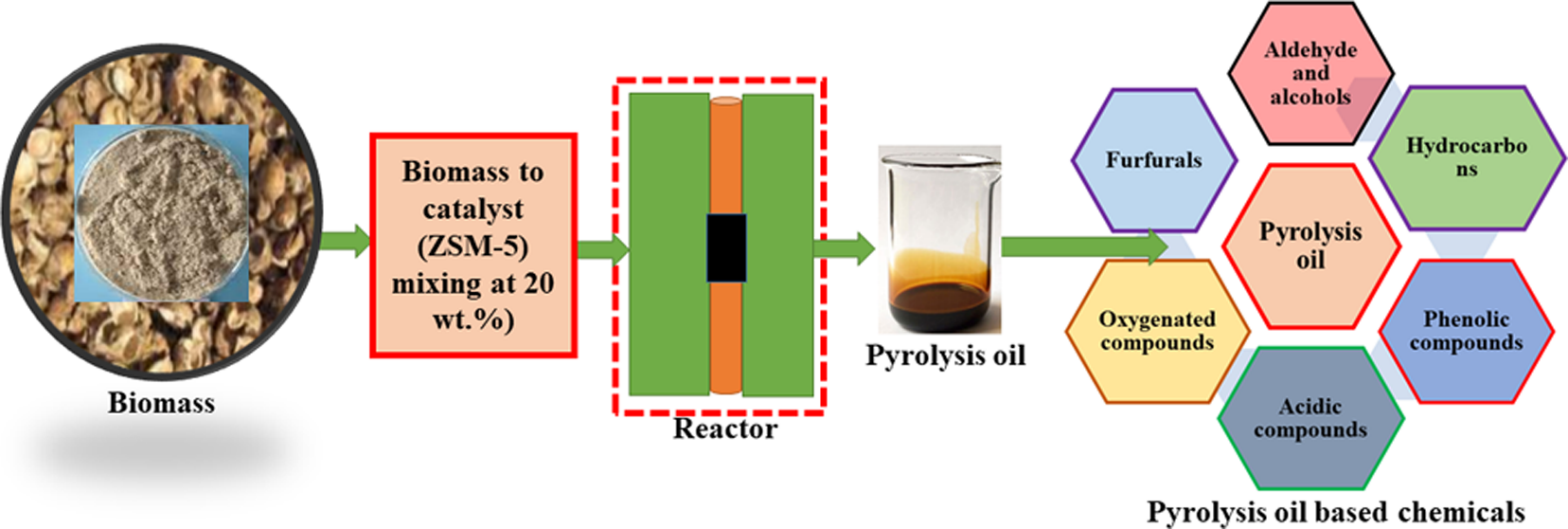

Pyrolytic oil is currently in its early stages of production and distribution but has the potential to grow into a significant renewable energy source. It may be processed into a variety of useful substances, including chemicals, and used for heating, transportation, and energy production. The present investigation involves the production and characterization of pyrolytic oil from areca nut husk (ANH), with and without ZSM-5. The pyrolysis experiment was conducted in a semibatch tubular reactor at 600 °C and a heating rate of 80 °C min^–1^ using ZSM-5 at 20 wt %. The pyrolytic oil was examined via elemental analysis, viscosity, density, moisture content, GC-MS, FTIR, higher heating value (HHV), and ash content. The analysis of kinetics verified that the activation energy rises in proportion to the conversion rate. Additionally, employing ZSM-5 in catalytic pyrolysis at 20 wt % boosted the yield of pyrolytic oil by 11% compared to thermal pyrolysis. Employing ZSM-5 at 20 wt % resulted in a decrease in viscosity, oxygen content, and density by approximately 43.40 cSt, 15.20%, and 168 MJ kg^–^1, respectively. Moreover, it led to an increase in higher heating value (HHV) and carbon content by 11.71 MJ kg^1–^ and 14.06%, respectively. An FTIR study of pyrolytic oil revealed the occurrence of hydrocarbons, aromatics, phenols, alcohols, and oxygenated chemicals. Moreover, GC-MS analysis indicated a significant increase in hydrocarbons (10.31%) and a decrease in phenols (2.36%), acids (6.38%), and oxygenated compounds with the introduction of the catalyst. Consequently, it can be inferred that utilizing ZSM-5 at 20 wt % during the pyrolysis of ANH aids in enhancing both the yield and characteristics of the resulting pyrolysis oil.

## 1 Introduction

The environment suffers from the widespread use of fossil fuels, but governments are becoming more committed to sustainable energy, which has sped up research on green fuels and other energy sources. The International Energy Agency (IEA) estimates that between 2007 and 2030, global energy consumption will increase by 1.50% yearly (from 12,000 to 16,800 Mtoe (million tonnes of oil equivalent)).^1^ In contrast, fossil fuels remain the primary energy source and are a significant factor in greenhouse gas (GHG) pollution and its effects, such as climate change and global warming. According to estimates from the Intergovernmental Panel on Climate Change (IPCC), greenhouse gases associated with fossil fuels are responsible for 56.60% of global greenhouse gas emissions.^1^ In light of this, the UN Climate Panel has set a goal of reducing such GHGs by 50 to 80% by 2050.^1^ To achieve this objective, it is imperative to reduce our dependence on fossil fuels and rapidly transition to renewable energy sources. These renewable energy sources have vast untapped energy capital to meet global energy demands at a lower cost than traditional fossil fuels. Among all renewable energy sources, biomass has become very well-known due to its environmental benefits.^2^ Dry plant matter, often known as biomass, is a low-cost renewable energy source that is widely accessible (220 billion tonnes a year).^3^ Committed and waste biomasses are considered viable and alluring fuel and energy sources. Lignocellulosic biomass (LBs) primarily comprises agricultural crop residues, aquatic plants, forestry waste, and other energy crops. These sources necessitate substantial processing and occupy considerable space when left unused. Consequently, repurposing waste byproducts into fuel and energy production not only alleviates waste disposal challenges but also enhances economic returns through comprehensive utilization across the entire production chain.^4^ Also, it is ideal for the growth of biobased economies, which entails the efficient use of biomass for fuel and power generation and the positive effects on jobs that follow.^4^

Biochemical methods (BCMs) and thermochemical methods (TCMs) are crucial pathways for converting biomass to sustainable fuel and valuable components. While the biological process requires a longer duration, thermochemical reactions can break down biomass within mere seconds or minutes.^5^ The major approved TCMs are hydrothermal liquefaction (HTL), gasification, combustion, and pyrolysis. However, the HTL is suited for wet and dry feedstocks. Pyrolysis has become more popular due to its diverse applicability. Pyrolysis is a thermal cracking route that breaks down organic materials in an air-deprived atmosphere at moderate temperatures (400–700 °C).^6^ Among thermochemical methods (TCMs), pyrolysis stands out as the sole technique capable of converting materials into solid, liquid, and syngas forms of energy simultaneously. The conversion of biomass into liquid fuel and char via pyrolysis is notably more efficient compared to other TCMs.^7^ Areca nut husks (ANH) are safe for the environment and are nontoxic. Further, ANH constitutes about 65–80% of the total weight and volume of the fruit.^8^ Areca nut husks are easily obtainable. The areca palm is grown mainly in Southeast Asia, India, and some parts of Africa. The availability of husks varies based on the size and breadth of areca nut production in a given location.

Prior to biomass being converted into fuel, conducting a kinetic study of the feedstock is essential. This expertise in the kinetics of the feedstock aids in optimizing process variables, designing innovative pyrolysis reactors, and simplifying mathematical modeling. Key kinetic parameters include the activation energy, frequency factor, and reaction order. Thermogravimetric analysis (TGA) stands out as the simplest analytical method for determining the thermochemical behavior of organic materials, facilitating the selection of suitable thermochemical methods (TCMs). The components approach, distributed activation energy model, and single-step, two-parallel reaction model^9^ are the TGA-reliant models utilized to identify the kinetic behavior of pyrolysis reaction mechanisms. Studying the kinetic behavior of lignocellulosic biomass can assist us in comprehending the workings of the pyrolysis reaction. To understand reaction behavior and optimize process parameters during the thermal deterioration of materials, kinetic parameters collected from the reaction are crucial. Also, it offers a chance to create effective thermochemical processes. TGA data analysis can be approached through various methods, primarily categorized into model-fitting and model-free methods for kinetic studies. Model-fitting techniques involve the use of different models to achieve the most suitable and optimal statistical fit for the data. In contrast, model-free methods do not rely on specific models for fitting. The model-free technique uses several heating rate curves to deliver kinetic parameters without making assumptions.^10^ Estimating complex material reactions may be possible using isoconversional (multiheating) techniques. The biggest benefit of model-free approaches is that there is no chance of choosing the incorrect kinetic model or determining the incorrect kinetic parameters.^11^ The isoconversional model is the foundation for the Kissinger–Akahira–Sunose (KAS), Ozawa–Flynn–Wall (OFW), Distributed Activation Energy Model (DEAM), and Vyazovkin method (VZ) models. These methods are included in the category of multiheating models. Isoconversional methods associated with many issues, such as the Flynn–Wall–Ozawa and Kissinger–Akahira–Sunose models, include their sensitivity to assumptions like constant heating rate, neglecting variations in activation energy, and potential inaccuracies in predicting reaction kinetics for complex reactions. Additionally, these methods may not provide detailed mechanistic insights and could be influenced by experimental errors or uncertainties in the data.

The potential of ANH to produce liquid fuel was revealed through an examination of its physicochemical characteristics and thermal pyrolysis.^1^ ANH’s physicochemical characteristics demonstrated its strong potential for replacing fossil fuels.^12^ It is necessary to use catalysts to improve the quality of pyrolysis oil (POs) since the thermal process that turns biomass into a liquid fuel output has various downsides, including high viscosity, oxygen acidity, and moisture content. Through catalytic pyrolysis, the reaction rate is increased, the yield is improved, and the product characteristics are improved.^13^ Moreover, proper catalyst application promotes transformation efficiency, lowers the rate of tars development, and raises the yield of the desired product.^14^ A variety of catalysts have been used to improve pyrolytic oil yield and properties, including metal oxide, zeolites, zeolite metal-based catalysts, etc. ZSM-5 has been widely used in the gas adsorption–separation and crude oil refinery industries due to its strong acidity and shape selectivity.^15^ The crystalline aluminosilicate material ZSM-5 has excellent temperature and hydrothermal stability, strong acid rigidity, resistance to carbon deposition, adjustable acidity, excellent shape selectivity, isomerization, hydro-deoxidization, and other catalytic properties.^16^ It also has a unique two-dimensional channel-like pore structure with intersecting channels of about 0.55 nm in diameter that favor hydrocarbons with about ten carbon atoms.^15^ ZSM-5 is commonly employed as a catalyst for biomass pyrolysis. Studies have found that it can substantially modify the composition of bio-oil by reducing oxygenated compounds through deoxidization reactions, enhancing aromatic compounds, and generating more organic matter (bio-oil) that can be upgraded for use in gasoline and diesel fuel.^15^ Scientists have reported their work to demonstrate the potential of ZSM-5 in biomass pyrolysis. Li et al. explored the effects of the desilication of ZSM-5 zeolite on its catalytic properties using beechwood powder in a semibatch reactor.^17^ They suggest that carefully managed zeolite desilication can enhance lignocellulose conversion to useful aromatic hydrocarbons and reduce the generation of undesirable coke, increasing the lignocellulose product distribution. Further, Li et al. explored the outcome of ZSM-5 on the pyrolysis of poplar sawdust using Py–GC/MS.^18^ The findings showed that the catalysts increased acidity and produced good monoaromatics and olefins selectivity. Further, hierarchical ZSM-5, which had a 4 wt % Fe loading, performed better than other models, with a 15.30% selectivity for monoaromatics.^18^ Nishu et al. explored the catalytic pyrolysis of rice straw-based lignin ZSM-5 in Py–GC/MS.^19^ They reported that ZSM-5 is the most suitable catalyst for the selectivity of hydrocarbons. Chen et al. studied the pyrolysis of cotton stalks over Fe-modified ZSM-5 and CaO in Py–GC/MS.^20^ They reported that 5Fe/CaO (10Fe/CaO) coupled with ZSM-5 promotes the formation of BTX. After a thorough examination of the existing literature, it was revealed that there have been no instances of pyrolyzing ANH using ZSM-5, whether in application or analysis. While there are a few studies on the pyrolysis of ANN, resulting in either biochar or kinetic investigations, the potential of ANH remains largely unexplored. This study aims to investigate the changes in product composition when ANH is subjected to pyrolysis with ZSM-5 at a concentration of 20 wt %. Further, a kinetic study of ANH over multiple heating rates using the VZ model is also missing in the literature. In addition, the biomass to catalyst loading also varied significantly, altering the pyrolytic oil properties and yield. As per the established literature, pyrolysis of ANH at 20 wt % of ZSM-5 at optimized conditions has not yet been studied to the best of the author’s knowledge. Furthermore, the process parameter also influenced the yield and properties of the pyrolytic oil. Therefore, the present work is focused on the kinetic decomposition of biomass at different heating rates (5, 15, 25 °C min^–1^) in a TGA. Further, the peak of the materials during TGA pyrolysis shifts, making long intervals unsuitable for generating precise findings. Because the higher heating rate is ineffective for these models, a lower heating rate was employed in this study. In addition, thermal and catalytic pyrolysis of ANH utilizing ZSM-5 at 20 wt % is performed as a result of the significant gap. The pyrolysis test was conducted in a cylindrical semibatch tubular reactor using ZSM-5 at 20 wt %. Moreover, pyrolytic oil was evaluated using elemental analyzers, viscosity, density, acidity, moisture content, gas chromatograph–mass spectrometry (GC-MS), and Fourier-Transform Infrared spectroscopy (FTIR).

## 2 Material and Methods

### 2.1 Sample Collection and Preparations

Areca nut husk (ANH) was received from the local village near Yeswanthpur (13.0250° N, 77.5340° E), Bangalore, India. ANH was washed with hot water (30–50 °C) to clean the dirt and unwanted Impurities. The washed sample was air-dried in an open environment for more than a week. Subsequently, it was subjected to further drying in a hot air oven at 60 °C for 3–5 h to ensure thorough removal of moisture and achieve uniform dryness. The uniformly dried biomass was ground into smaller particle sizes (<1 mm) using a moisture grinder and sieved. The grounded sample was placed in an airtight container to avoid moisture adsorption. Further, the sample is dried before the experiment to prevent moisture impurities. ZSM-5 (SI/Al = 25) was purchased from Merck and Co., Inc. India and used with biomass without treatment.

### 2.2 Characterization of Biomass

The bioenergy potential of biomass can be demonstrated through proximate and elemental analysis. Proximate analysis of ANH involved moisture content analysis conducted according to ASTM D3173-11, volatile matter analysis following ASTM-D3175, and ash content analysis using ASTM D2584. Elemental analysis of ANH and ANH-derived pyrolysis oil (ANHPO) was carried out using a PerkinElmer elemental analyzer (Thermo Scientific Flash 2000). Additionally, the bulk density of ANH was determined by using a digital balance and graduated cylinder. The sample mass was determined with a digital weight balance, while the sample volume was measured using a graduated cylinder. The higher heating value (HHV) of ANH and pyrolytic oil was calculated using a plain jacket oxygen bomb calorimeter (Parr Instruments, Model 1341). The extractive content of ANH was analyzed using a Soxhlet apparatus. Five grams of dry-weight ANH sample was placed in a cellulose thimble, which was then inserted into a Soxhlet tube. To a round-bottom flask, 250 mL of solvent (hexane) was added. The Soxhlet apparatus was heated using a heating mantle at 70 °C for 5 h. After this period, the sample was allowed to cool and then dried in a hot air oven at 60 °C for 3 h. Subsequently, the same sample was subjected to Soxhlet extraction, using ethanol as the solvent. The Soxhlet tube was heated at 79 °C for 5 h. The difference in weight before and after extraction was used to calculate the extractive content. Finally, the biochemical composition of ANH was determined using wet chemistry methods.^21^

### 2.3 Thermal Stability Analysis

The weight loss profile of ANH was analyzed by using a thermogravimetric analyzer (NETZSCH, TG 209 F1 Libra) within an oxygen-deprived environment. 8 ± 0.2 mg of sample was placed in the analyzer and heated from 30 to 900 °C at a heating rate of 10 °C min^–1^, with a sweeping gas flow rate of 50 mL min^–1^. Additionally, using the same TGA setup and conditions, the dynamic thermal behavior of ANH was examined at three different heating rates: 5, 15, and 25 °C min^–1^. To ensure data reliability, the TGA experiment was repeated three times.

### 2.4 Fourier-Transform Infrared Spectroscopy (FTIR) Analysis

A Shimadzu (Model No.: IRAffinity-1) was used to conduct an FTIR analysis of ANH and pyrolytic oil (ANHPO) to categorize the attendance of the functional group. The dried powdered/liquid sample was loaded into the attenuated total reflectance (ATR), which ran in the 400–4000 cm^–1^ range with a step size of 4 cm^–1^ and a 40 scan-per-second scanning rate.

### 2.5 Kinetic Theory and Thermodynamic Analysis

Biomass is one of the multifaceted biopolymers, and its constitution varies from location to location. It is nearly hard to imagine an ideal reaction mechanism because several sorts of reactions occur during pyrolysis in a fraction of a second. The following equations, shown in Table 1, were used to compute biomass’s kinetic parameters.

**Table 1:** Kinetic Model was Used in This Study to Estimate the Kinetic Parameters^a^

model name	equation
Kissinger–Akahira–Sunose (KAS)	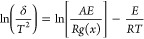 1
Ozawa–Flynn–Wall (OFW)	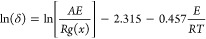 2
Distributed Activation Energy Model (DAEM)	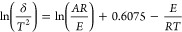 3
Vyazovkin model (VM),	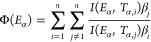 4
	The temperature integral is given as.
	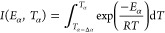 5

a where *n, T, β, R, A, E*, and h are denoted as the reaction order, absolute temperature (K), heating rate (°C min^–1^), gas constant (J mol^-1^. K^-1^), pre-exponential factor (min^–1^ or s^–1^), activation energy (kJ mol^–1^), peak decomposition temperature (K), and Plank constant 6.626 × 10^–34^ Js, respectively.

### 2.6 Pyrolysis Setup and Experiments

**Figure 1:**
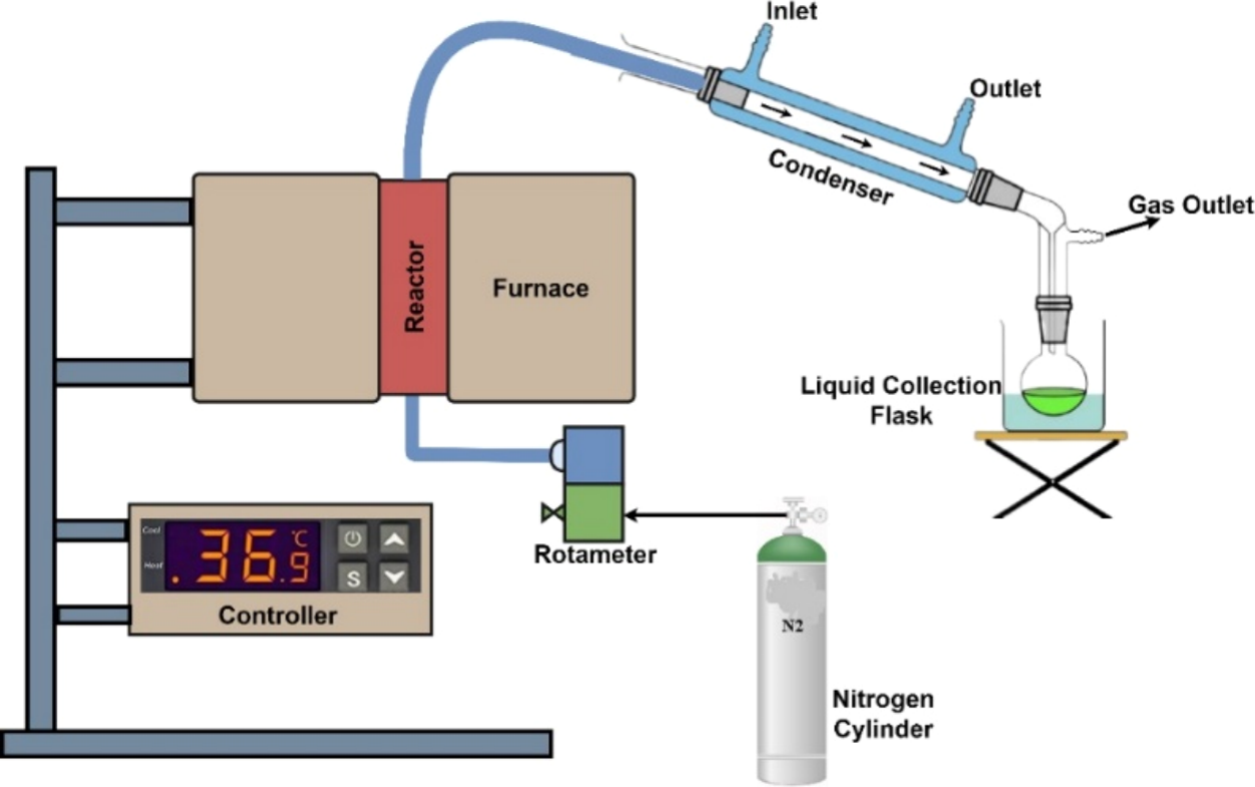
Schematic layout of the experimental setup.


A stainless steel (SS-304) cylindrical semibatch reactor with dimensions of an 8 cm internal diameter (ID), 7.60 cm outer diameter (OD), and 40 cm in length was used for the pyrolysis test. The control panel, thermocouple, condenser, water chiller, nitrogen gas cylinder, and rotameter represent most of the investigational setup. A predetermined amount of dry sample (500 g) was added to the reactor and then positioned vertically within the ceramic brick-built furnace. Heat is dispersed evenly throughout the investigation thanks to the reactor’s design. A PID controller mounted on the control panel regulated the temperature, residence duration, and heating rate. Further, a K-type thermocouple was inserted in the furnace, which is directly associated with the reactor and is used to measure the temperature. The reactor was installed within the furnace to ensure a uniform distribution of heat across its entirety. Additionally, nitrogen gas was circulated for 15 min before commencing the tests to eliminate any undesired contaminants from the reactor. During the pyrolysis process, the flow rate of nitrogen gas was regulated by the rotameter. The nitrogen gas inlet was connected to the bottom end of the reactor, while the top end was linked to the condenser. To maintain optimal conditions, a water chiller was employed to recirculate cold water (maintained at 8–10 °C) within the condenser throughout the trials. When the noncondensable gases were expelled from the storage tanks, the condensable gases were condensed in the condenser and stored. Finally, the reactor was cooled to room temperature (30 °C) before the biochar was obtained, and the yields of the liquid, char, and syngas were calculated using eqs 6, 7, and 8. Figure 1 depicts the complete laboratory investigational setup.

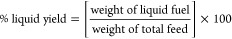
6
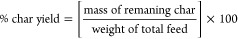
7
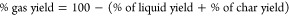
8

The pyrolysis experiment was conducted in a semibatch tubular reactor at 600 °C, with a heating rate of 80 °C min^–1^ and a nitrogen flow rate of 100 mL min^–1^. The blending of ANH and ZSM-5 was performed manually with a loading of 20 wt %. This loading refers to 20 wt % of the total weight of biomass used in the mixture. The previous study confirmed that out of 10, 20, and 30 wt % ZSM-5 loading, 20 wt % provides higher liquid yield with improved properties.^22^

### 2.7 Characterization of Pyrolytic Oil

The pyrolytic liquid was left overnight in a separating funnel to facilitate the separation of organic and aqueous phases based on their density difference. The organic oil, termed pyrolytic oil, rose to the top layer, while the bottom layer consisted of the aqueous phase. The present study only characterizes pyrolytic oil. Using the HAAKE RheoStress 1, Cone (Cup Z 43 (Series 1)), and Plate (PP 35 Ti, *D* = 35 mm) types of geometries at 30 °C and 50 rpm, the viscosity was calculated. Karl Fischer’s water analyzer was used to measure the moisture (Metrohm 787 KF Titrino). Also, an Eutech waterproof (pH Spear) pH meter was used to check the acidity; nonetheless, a density meter was used to check the density (Anton Paar) of pyrolytic oil. A density meter was filled with 1 mL of organic oil devoid of air, and the average values were recorded. The higher heating value (HHV) of pyrolytic oil was measured by using an oxygen bomb calorimeter (1341 Plain Jacket Calorimeter). Further, the DIN EN-7 standard was also used to assess the pyrolytic oil’s ash content. A hot air oven set at 105 °C for 1 h was used to eliminate the moisture content of the pyrolytic oil. One gram of dried pyrolytic oil was placed in a ceramic crucible that had been dried, weighed, and heated for 24 h at 775 °C. Once the experiment was completed, the sample was taken out of the muffle furnace and transferred to a desiccator for isothermal cooling. The variance between the initial and final weights reveals the amount of ash present in the pyrolytic oil.

### 2.8 GC-MS Analysis

The organic oil was analyzed using a Gas Chromatograph–Mass Spectrometer (Varian, 450-GC, 240-MS; Netherlands). Elite 5 MS column (diameter 0.250 mm, length 30 m) was used to identify the hot vapor. Throughout the analysis, He gas with a 99.99% transparency was supplied as a carrier gas at a flow rate of 1 mL min^–1^. Moreover, 1 μL of the sample was added after diluting with dichloromethane (DCM) and pyrolytic oil at 100:1 (vol/vol). To allow for the extraction of all constituents, the GC was configured to start at 40 °C for 1 min before ramping up to 280 °C at 5 °C min^–1^ and holding for 15 min. Injector, interface, and MS ion source temperatures were maintained at 280, 280, and 250 °C, respectively. The electron ionization voltage was held at 70 eV while the split injector ratio remained at 10:1. By comparison of the acquired mass spectra with the National Institute of Standards and Technology (NIST) collection, the unidentified products discovered in the organic oil were identified.

## 3 Results and Discussion

### 3.1 Physicochemical Characterization of ANH

**Figure 2:**
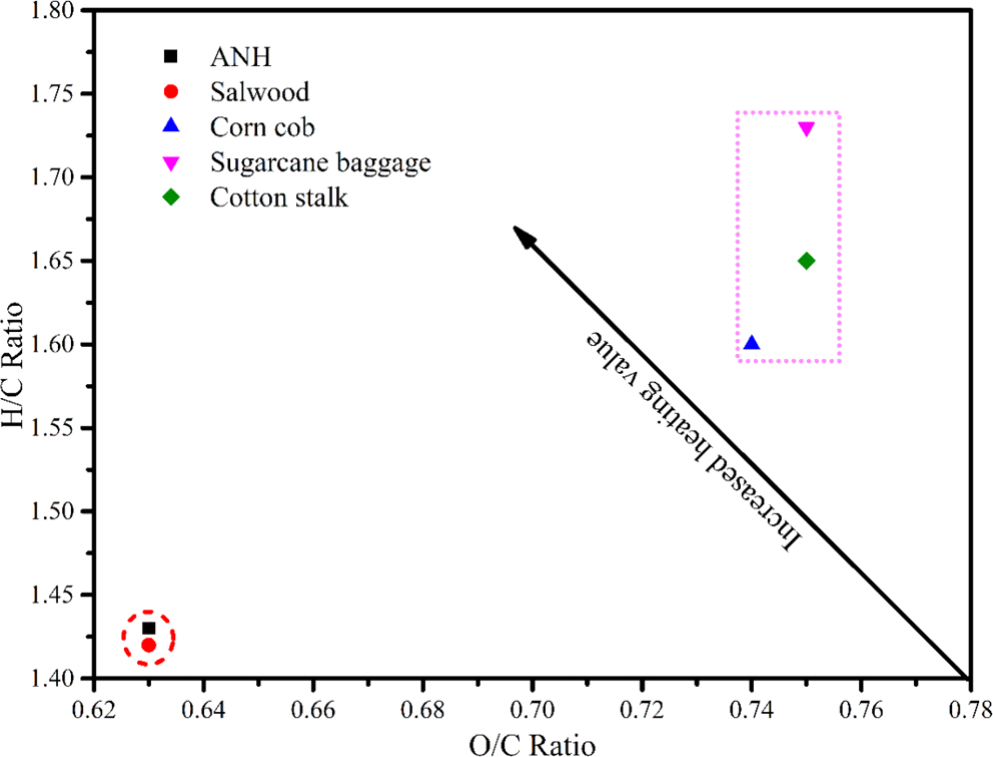
Van-Krevelen diagram of ANH.

Table 2 lists the physicochemical characteristics of ANH that govern its applicability. The results are also contrasted with data from previous peer-reviewed studies on sal wood sawdust,^23^ cotton stalk,^24^ sugar cane baggage,^24^ and corn cob.^24^ According to the close investigation, ANH was found to be 6.21% moisture, 76.43% volatile matter, 2.37% ash, and 14.99% fixed carbon. In comparison to other feedstocks for pyrolysis, ANH was discovered to possess a moisture content of 6.21%, which fell below the permissible limits (>10%). In addition, the volatile matter (76.43%) is found to be equivalent to sugar cane baggage and sal wood sawdust. However, the volatile matter of ANH was found to be lower than corn cob (80%) and higher than cotton stalk (71%), respectively. Further, the ash content of ANH is found to be 2.37%, which is lower than that of the cotton stalk (3.50%), sugar cane baggage (4.40%), and corn cob (5.70%) and equivalent to that of sal wood sawdust (2.02%) (Table 2). Since ANH has a lower ash content and higher volatile content, it is easier to ignite during combustion.^25^ Furthermore, the higher ash content in biomass acted as a heat sink, thereby reducing its heating value. This effect led to a significant decrease in fouling and slagging within the boiler or furnace, thanks to the lower ash level.^26^ Given the diverse biochemical and structural composition of biomass, the fixed carbon content was determined to be 14.99%. This value falls slightly above that of corn cob and sugar cane bagasse yet below that of sal wood sawdust and cotton stalk (Table 2). ANH has 50.12% carbon, 6% hydrogen, 42.76% oxygen, and 1.12% nitrogen; however, sulfur content is found to be absent. The greater carbon content and lower hydrogen in biomass confirmed that it would likely have an HHV and potential for energy production.^27^ Additionally, the reduced oxygen level offers the advantage of enhancing the yield of high-quality bio-oil, as higher oxygen content tends to diminish the heating value of the fuel. Furthermore, the lower levels of nitrogen and sulfur in ANH resulted in a significant reduction in the levels of the generation of SOx and NOx during pyrolysis. It is possible to visualize the energy density of feedstock using the Van-Krevelen diagram (VKD). The atomic composition of biomass is being studied by VKD. Sal wood sawdust,^23^ cotton stalk,^24^ sugar cane baggage,^24^ and corn cob^24^ all have an atomic ratio with ANH, and VKD is displayed in Figure 2. The hydrogen/carbon ratio (H/C) of 1.43, as determined by the results of the elemental investigation, is lower than that of the cotton stalk, sugar cane baggage, and corn cob and higher than that of sal sawdust. Also, it was discovered that all of the samples shown in Table 2 and Figure 2 have an oxygen to carbon (O/C) ratio that is lower than that of stalk, sugar cane baggage, and corn cob and that is equivalent to sal wood sawdust. The alteration in the atomic ratio of all of the material was produced by its metabolic composition. The carbonaceous fuel’s atomic composition confirms the fuel’s igniting effectiveness.^28^ Moreover, to achieve a higher fuel grade, a fuel’s atomic ratio (H/C) must be more significant than its atomic ratio (O/C).^28^ Further, ANH has a heating value (HHV) of 18.44 MJ kg^–1^ and a bulk density of 3.71.23 kg m_,_^–3^ which indicates that transportation and storage would be easy. The ANH bulk density and HHV values are in good agreement with the other biomass data in Table 2. The extractive percentage of ANH was found to be 13.17 wt % by using Soxhlet equipment. During pyrolysis, a greater extractive concentration encouraged the production of more liquid products. Following other biomass mentioned in Table 2, the biochemical composition of ANH indicated 16.81% hemicellulose, 48.48% cellulose, and 13.17% lignin. It is essential to keep in mind that different types and locations of biomass have different contents. The outcomes of the physical and chemical investigations are in agreement with the remaining biomass listed in Table 2.

**Table 2:** Physicochemical Characterization of ANH and Comparison with Other Testified Biomass

analysis	Areca nut husk (ANH)	sal wood sawdust^23^	cotton stalk^24^	sugar cane baggage^24^	corn cob^24^
Proximate Analysis (wt %, Dry Basis) (Dry Basis)					
moisture content	6.21 ± 0.10	6.04 ± 0.20	8.9	10.0	10.2
volatile matter	76.43 ± 0.20	76.03 ± 0.10	71.0	76.0	80.0
ash content	2.37 ± 0.05	2.02 ± 0.01	3.5	4.4	5.7
fixed carbon	14.99 ± 0.30	15.99 ± 0.20	16.6	9.6	4.2
Ultimate Analysis (wt %) (Dry Basis)					
C	50.12 ± 0.10	50.43	46.8	43.2	44.2
H	6.0 ± 0.10	5.99	6.4	6.2	5.9
O	42.76 ± 0.12	43.06	46.8	43.2	44.2
N	1.12 ± 0.006	0.52	0.3	0.4	0.54
S			0.2	0.8	0.08
O/C	0.64	0.63	0.75	0.75	0.74′
H/C	1.40	1.42	1.64	1.73	1.59
heating value (MJ/kg)	18.44 ± 0.16	19.18 ± 0.19	19.2	17.2	15.5
bulk density (kg/m^3^)	371.23 ± 10	330.12 ± 20			
chemical analysis (wt %)	78.46	78.95	81.10	75.1	77.0
hemicellulose (HC)	16.81 ± 1.12	16.23	19.2	18.7	29.0
cellulose (C)	48.48 ± 1.24	49.52	39.4	36.6	32.2
lignin (*L*_g_)	13.17 ± 1.16	13.20	23.2	19.8	15.8
extractive content (wt %)	9.79 ± 0.18	11.23	7.6	19.4	14.8
hexane/water	8.12 ± 0.11	10.02 ± 0.12	6.2	17.2	12.3
ethanol	1.85 ± 0.02	1.21 ± 0.11	1.4	2.2	2.5

### 3.2 FTIR Analysis

The intricate interactions among water, phenols, acid, alkane, aliphatic, and aromatic components were seen in the ANH FTIR spectra. Figure 5, section 3.6, shows a spectrum with wavenumber and transmittance shown against each other. The presence of water, acids, phenols, proteins, and aromatic components was demonstrated through the absorption band of −OH deformation, observed at 3452 cm^–1^.^29^^29^ Alkane and carbonyl/carboxylic acid were found in the absorption band 2892 cm^–1^, which was linked to C–H stretching.^30^ Alkyne was proven to exist by peaks in the region of 1232–1455 cm^–1^ associated with C–C deformation vibration, while the survivability of aromatics and alkene was hindered by the adsorption band 1642 cm^–1^ attributable to C=C stretching vibration.^30^ The peak region below 1000 cm^–1^ was attributed to O–H bending, suggesting the prevalence of mono- and polycyclic-substituted aromatic components, while the adsorption band at 1038 cm^–1^ was connected to C=O stretching and deformation vibration, revealing the distribution of esters and ether.^29^

### 3.3 Thermal Decomposition Variation of ANH against Heating Rates

**Figure 3:**
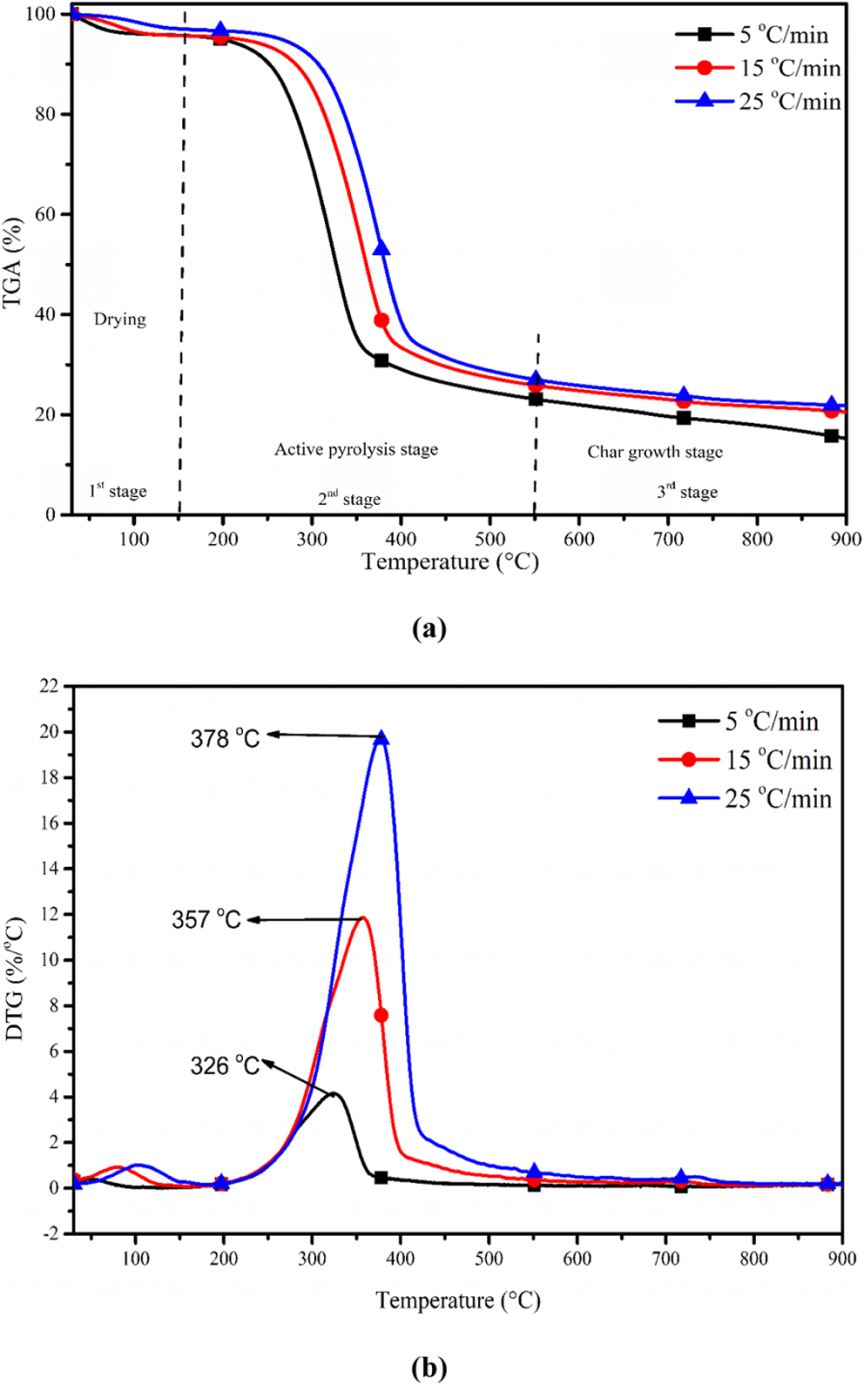
Variation of the thermal profile of ANH against heating rate: (a) TGA and (b) DTG.

The breakdown curve of ANH as a function of the temperature is shown in Figure 3. ANH underwent decomposition in three primary stages: dehydration (up to 150 °C), active pyrolysis (150–550 °C), and char formation (>550 °C). During the initial phase, predominantly light volatile compounds and chemically bound moisture were released. However, the temperature range between 150 and 550 °C exhibited the most significant breakdown of ANH. In the second step of biomass decomposition, the principal components, hemicellulose and cellulose, undergo breakdown into polymers with lower molecular weights under continued heat input. It is widely agreed upon that the onset of biomass dissociation, which results in the production of tarry odors that persist, typically occurs around 170 °C.^31^ Similar results are found in the current experiment, with slight variances caused by variations in chemical composition. The components of biomass were spread over the lower and intermediate phases. Moreover, hemicellulose has a larger moisture level than lignin because it has more hydration.^32^ Pentosan hydrolysis and dehydration have reduced the thermal stability of xylan, the last ingredient of hemicellulose disintegration.^33^ Xylan, a complex sugar polymer, serves as a significant constituent of hemicelluloses found in plant cell walls. When hemicellulose undergoes disintegration, xylan is broken down into xylose or other sugar monomers through processes like hydrolysis or enzymatic degradation.^34^ In contrast to hemicellulose, cellulose is broken down in three steps. The initial stage involved lower temperatures (280 °C), and it included interactions that led to cellulose depolymerization via bond cleavage, dehydration, the establishment of oxidants (carbonyls, carboxyl groups, and peroxides), the advancement of free radicals, the appearance of CO and CO_2_, and subsequently biochar expansion.^33^ The following stage occurs between 280 and 550 °C, producing tar-rich pyrolysates (anhydrosugars, levoglucosan, and oligosaccharides) and small glucose from glycosidic bond depolymerization. The last explosion stage then causes the direct breakdown of cellulose into simpler molecules and hot volatiles through fission, dehydration, disproportionation, and decarboxylation reactions at a temperature of over 550 °C.^33^ Lignin exhibits remarkable thermal resistance, fracturing at significantly high temperatures, typically exceeding 550 °C. The decomposition of lignin typically initiates within a broad temperature range spanning from 150 to 900 °C.^35^ The dissolution of lignin is ascribed to the destruction of C–O bonds, ultimately forming molecules containing only one oxygen atom. The methoxy cleavage of C–O bonds at 327–380 °C produces products with two oxygen atoms after that, and finally, the chain length C–C links between carbon particles and aromatic rings split. Breaking the weaker linkages (alkyl-aryl ether) under mild reaction conditions provides the basis for the creation of biochar.^36^ The opening peak (∼70 °C) that emerged in the early phase was generated by the elimination of chemically bound humidity and deficient hot volatiles, according to DTG thermographs of ANH depicted in Figure 3.^37^ Additionally, the disintegration of hemicellulose and cellulose by a heat supply resulted in the peaks in the second stage at 323 °C. Finally, lignin breakdown occurred at a high temperature (>550 °C), slowly and without any abrupt peaks.^37^ Since lignin breaks down more slowly than hemicellulose and cellulose, there are no noticeable peaks because of the lower lignin fraction.^38^ According to the thermal study of ANH, it was observed that 6.70% of the biomass decomposed in the first stage, 69.90% in the second stage, and 7.95% in the third stage.

The impact of heating rates on ANH at dynamic heating rates (5, 15, and 25 °C min^–1^) is depicted in Figure 3a,b. TGA thermographs (Figure 3a) were shown to shift at higher temperatures (326, 357, and 378 °C) as heating rates increased from 5, 15, and 25 °C min^–1^, respectively. It was clear that biomass is a poor heat conductor, which caused a temperature boundary to build throughout the particle’s outermost layer during thermal implementation.^39^ Other potential causes include the development of secondary reactions during the disintegration of tar and heavier mass.^39^ The faster pyrolysis is more effective than slower pyrolysis since the biomass fractured more swiftly and heated volatiles left the reactor more swiftly (reducing the residence time).^40^ The various pyrolysis techniques and other process variables directly impacted the targeted outputs. Also, it was observed that the degradation temperatures of specific compounds such as hemicellulose, cellulose, and lignin altered with increased heating rates. The second peak of the cellulose degradation process proceeded at 326, 357, and 378 °C, respectively (Figure 3b). Also, a slight fluctuation in the total volatile transformation (69, 70.08, and 72.68%) in the second phase was seen with increases in heating rates.

### 3.4 Kinetic Study

Table 3 (a) lists the derived results from the kinetic studies of ANH using KAS, OFW, DAEM, and VZ, respectively. Due to a lower value for the correlation coefficient, a conversion value higher than 0.8 did not match the data well (Table 3 (a)).^41^ It was found that the apparent activation energy (AAE) increased with an increasing conversion value. Our results are consistent with other listed literature studies.^30^ According to the KAS, OFW, DAEM, and VZ, ANH’s average apparent activation energy (AAAE) values are 209.02, 221.38, 169.94, and 234.37 kJ mol^–1^, respectively. The coefficient of correlation (*R*^2^) was greater than 0.9 for every model at each conversion value, indicating that the chosen model fit the experimental results. Also, it was observed that the activation energies rarely changed with the conversion rate, indicating that a greater degree of reaction might have occurred in a single step.^42^ Further, the AAE varied from 169.64 to 263.19, 175.91–295.64, 171.27–180.23, and 182.66–349.81 kJ mol^–1^, respectively, for KAS, OFW, DAEM, and VZ. Furthermore, the frequency factor varies from 2.88488 × 10^17^–1.62904 × 10^21^, 9.60279 × 10^17^–2.98185 × 10^23^, and 7.12476 × 10^12^–1.00053 × 10^11^ min^–1^, respectively, for KAS, OFW and DAEM. The pyrolysis reaction mechanism’s function is the activation energy. As activation energy plays a prominent role in reactions, larger activation energy values indicated slower reaction rates and lower activation energy values indicated faster reaction rates.^41^ The activation energy of a fuel can be used to estimate its reactivity throughout pyrolysis and combustion.^43^ The developed pyrolyzer and process parameter optimization exploits the derived activation energy. Further, the error analysis test of AAE confirmed the following error trend: KAS> OFW > DAEM > VZ. Therefore, it can be supposed that using DAEM and VZ for kinetic parameter investigation produces minimal error.

**Table 3 (a):** Kinetic Analysis of Biomass Using KAS, OFW, and DAEM Models

model	conversion (*x*)	*E* (kJ/mol)	*A* (1/min)	*R*^2^	fitting equation
KAS	0.1	169.64	2.88488 × 10^17^	0.9039	*y* = −20404*x* + 27.408
	0.2	186.02	5.15807 × 10^16^	0.9676	*y* = −22375 + 29.048
	0.3	190.52	1.42918 × 10^18^	0.9808	*y* = −22916*x* + 28.862
	0.4	189.32	4.84431 × 10^17^	0.9839	*y* = −22771*x* + 27.735
	0.5	195.48	7.98602 × 10^17^	0.9876	*y* = −23515*x* + 28.198
	0.6	205.64	2.84717 × 10^18^	0.9896	*y* = −24734 + 29.431
	0.7	272.37	3.26178 × 10^23^	0.9508	*y* = −32761*x* + 41.032
	0.8	263.19	1.62904 × 10^21^	0.9994	*y* = −31657*x* + 35.536
	average	**209.02**			
OFW	0.1	175.91	9.60279 × 10^17^	0.9203	*y* = −21159*x* + 41.406
	0.2	195.50	9.90925 × 10^18^	0.9709	*y* = −23515*x* + 43.74
	0.3	200.25	806752 × 10^18^	0.9709	*y* = −24087*x* + 43.607
	0.4	199.27	2.9343 × 10^18^	0.9855	*y* = −23968*x* + 42.523
	0.5	205.62	4.83784 + 18	0.9888	*y* = −24732*x* + 43.023
	0.6	215.95	1.72268 × 10^19^	0.9906	*y* = −25975*x* + 44.293
	0.7	282.95	1.97577 × 10^24^	0.9543	*y* = −34033*x* + 55.943
	0.8	295.64	2.98185 × 10^23^	0.9994	*y* = −35559*x* + 54.052
	average	**221.38**			
DEAM	0.1	171.27	7.12476 × 10^12^	0.9996	*y* = −20601*x* + 26.982
	0.2	163.19	3.51703 × 10^11^	0.9992	*y* = −19629*x* + 24.02
	0.3	163.72	1.72958 × 10^11^	0.9991	*y* = −19693*x* + 23.301
	0.4	166.26	1.49544 × 10^11^	0.999	*y* = −19998*x* + 23.146
	0.5	168.55	1.31657 × 10^11^	0.9991	*y* = −20274*x* + 23.005
	0.6	170.57	1.09748 × 10^11^	0.9992	*y* = −20517*x* + 22.812
	0.7	175.73	1.64555 × 10^11^	0.9987	*y* = −21137*x* + 23.187
	0.8	180.23	1.00053 × 10^11^	0.9981	*y* = −28871*x* + 33.471
	average	**169.94**			

The DAEM showing lower activation energy compared to the KAS and OFW models could be attributed to differences in the underlying assumptions or methodologies of these kinetic models.^44^ The activation energy in chemical kinetics represents the energy required for a reaction to proceed. In the case of DAEM, its model might consider a distribution of activation energies, allowing for a more nuanced representation of the reaction mechanism. This broader perspective may account for variations in energy requirements across the reaction process, resulting in an overall lower average activation energy.^44^ Conversely, KAS and OFW models might make simplifying assumptions or rely on different mathematical expressions that lead to higher calculated activation energies. Further, the lower activation energy predicted by the Vyazovkin model compared to that of the Distributed Activation Energy Model (DAEM) may arise from the distinct mathematical approaches and assumptions inherent in each model. Vyazovkin’s model is known for its flexibility in handling diverse kinetic scenarios, potentially incorporating specific features or assumptions that lead to a lower calculated activation energy.^45^ The variations in the representation of reaction mechanisms, treatment of uncertainties, or consideration of particular kinetic parameters could contribute to the differences in activation energy predictions (Table 3(b)).

**Table 3(b):** Kinetic Study of Biomass Using the VZ Model

model	conversion (*x*)	*E* (kJ mol^–1^)	error
VZ	0.1	182.66	0.0244
	0.2	187.31	0.0036
	0.3	187.86	0.0017
	0.4	195.75	0.0017
	0.5	203.13	0.0017
	0.6	220.52	0.0037
	0.7	347.97	0.6051
	0.8	349.81	0.6700
	average	**234.37**	

### 3.5 Characterization of Pyrolytic Oil

**Figure 4:**
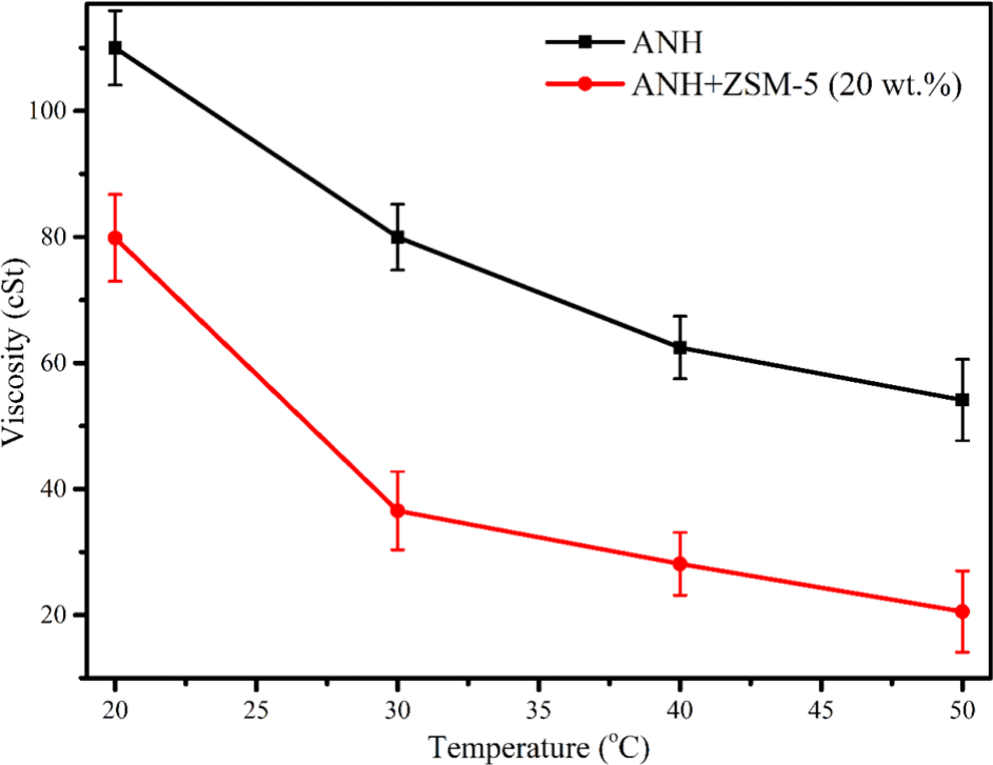
Variation of viscosity against temperature.

The yield of pyrolysis oil derived from pyrolysis of ANH with ZSM-5 at 20 wt % is reported in Table 4. The presence of a catalyst was observed to enhance the pyrolysis yield, likely due to increased synergistic interaction between biomass and the catalyst. Table 4 provides a summary of the characteristics of both thermal and catalytic pyrolytic oil, along with a comparison of diesel and gasoline.^46^ The yield of biochar from thermal pyrolysis was determined to be 29.56 ± 2.72 wt %, while catalytic pyrolysis resulted in a yield of 34.45 ± 1.51 wt %. It is essential to mention that weight was included while calculating the char yield catalysts. The catalytic pyrolytic oil had a significantly higher carbon content (73.43%) than thermal oil (59.36%) and a lower oxygen content (15.44%) than thermal oil (30.64%). Because the oxygen interaction during catalytic pyrolysis causes hydrogen and water to react, this process has the highest possible watery fraction. Also, due to a rise in the H/C ratio, catalytic pyrolysis boosted the liquid fuel’s heating value (38.16 MJ kg^–1^) compared to thermal pyrolysis (26.45 MJ kg^–1^). Thermal pyrolysis oil has a higher viscosity (79.96 cSt), which is reduced significantly by the catalytic pyrolytic oil (36.56 cSt). The stability of the pyrolytic oil is impacted by the higher viscosity, which also reduces its fluidity. A gain in molecular weight may be associated with the pyrolytic oil’s rising viscosity over time if the volatile is kept from escaping during storage.^46^ Unsaturated oxygen may cause polymerization reactions in the pyrolytic oil, which might increase the viscosity. Figure 4 depicts the variation of viscosity against temperature. It was found that increasing temperature decreased the viscosity of thermal and catalytic pyrolysis oil. The viscosity decreases as temperature rises because particles can more easily fix the adhesion forces, keeping them connected since they have greater thermal energy.^47^ The most effective catalyst for increasing the viscosity and heating value of the bio-oil was discovered to be ZSM-5.^48^ Moreover, the pyrolytic oil’s moisture content rises over time due to water production during condensation polymerization activities. However, by lowering the oxygen concentration, catalytic pyrolytic oil (ZSM-5 at 20 wt %) greatly decreased the viscosity of the pyrolytic oil (36.56 cSt). Catalytic pyrolytic oil has a greater moisture content (3.20%) than thermal oil (2.10%), which arises due to the interaction between oxygen and hydrogen molecules. Thermal pyrolytic oil has a higher density (980 kg m^–3^) than catalytic oil (812 kg m^–3^), making it suitable for transportation and storage and helping in the blending process. Because the acidity of thermal pyrolytic oil is lower (6.89) than that of catalytic pyrolytic oil (7.21), the heating value of the fuel is raised by increasing the H/C ratio. Thermal pyrolytic oil has a lower ash content (0.37%) than catalytic pyrolytic oil (0.43%). High ash content in bio-oil can lead to increased combustion-related emissions and the formation of ash deposits, negatively impacting its combustion efficiency and overall environmental performance.^49^ This might be because solid char or aromatic compounds are present, which produce smoke and leave behind more carbon residue. These qualities of pyrolytic oil increased its appeal as a fossil fuel substitute.

**Table 4:** Physicochemical Characterization of Pyrolysis Oil^a^

analysis	thermal	ANH + ZSM-5 (20 wt %)	diesel	gasoline
color	black and brown	black and brown	light yellow	
yield (wt %)	38.62 ± 1.61	49.63 ± 2.11		
C (%)	59.36 ± 1.02	73.42 ± 1.41	87.40	
H (%)	7.52 ± 0.86	9.08 ± 1.11	12.10	
O (%)	30.64 ± 1.50	15.44 ± 2.41		
N (%)	1.89 ± 0.21	1.56 ± 0.82	392 (ppm)	
S (%)	0.59 ± 0.06	0.5 ± 0.02	1.39	
HHV (MJ/kg)	26.45 ± 0.12	38.16 ± 0.16	45.50	47.30
viscosity at 30 °C at 30 rpm (cSt)	79.96 ± 0.42	36.56 ± 0.21	2.00–4.50	0.12
density (kg/m^3^)	980.0 ± 4.49	812.0 ± 2.89	828.00	838.00
moisture (%)	2.10 ± 0.15	3.2 ± 0.15		<0.10
acidity	6.89 ± 0.07	7.21 ± 0.04	5.5–8.0^a^	
carbon residue (%)	0.37 ± 0.02	0.43 ± 0.04		

a Standard data taken.

### 3.6 FTIR Analysis of Pyrolytic Oil

**Figure 5:**
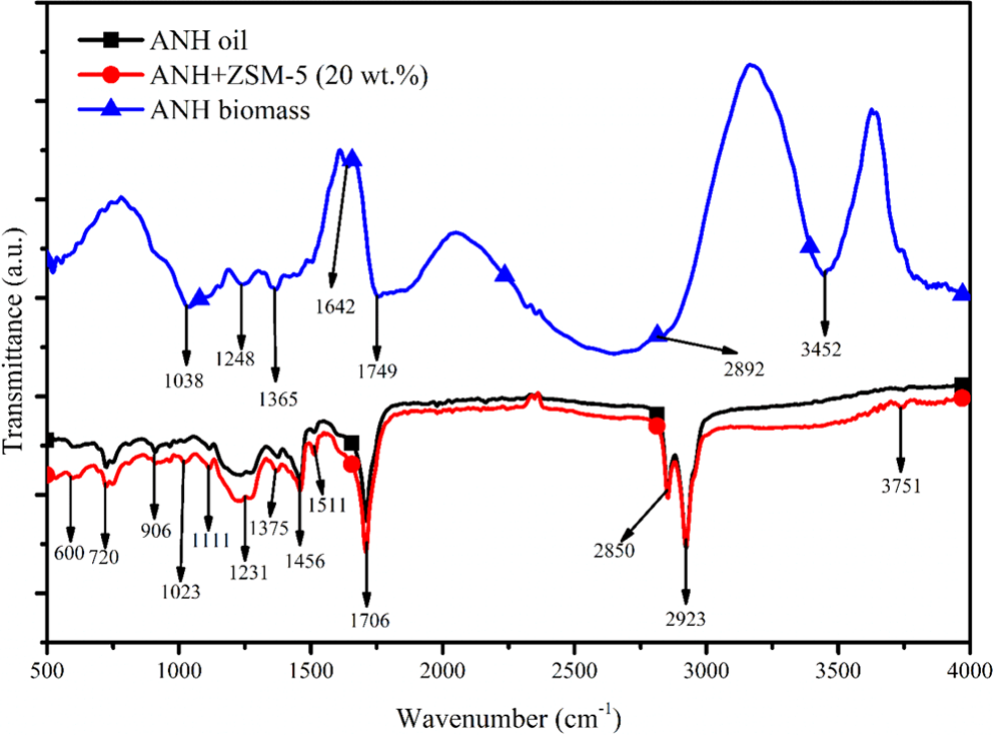
Functional group study of pyrolysis oil using FTIR.

Raw biomass, thermal, and catalytic pyrolytic oil were examined using Fourier-transform infrared spectroscopy (FTIR) to identify the existence of the beneficial functional group. Figure 5 displays the FTIR thermal and catalytic pyrolytic oil spectra. The FTIR spectrum’s 3864–3193 cm^–1^ adsorption band, which is linked to the −OH group, shows the presence of water, phenols, alcohol, and aromatics.^29,50^ Furthermore, the occurrence of saturated aliphatic and alkane groups was confirmed by peak 2923 cm^–1^, which was related to C–H stretching vibration (also supported by GC-MS results), whereas peak 2850 cm^–1^ showed an abundance of alkanes.^51^ Further, peak 1706 cm^–1^ revealed the availability of aldehyde, ketone, and ester due to C= O stretching vibration^51^ (supported by GC-MS results). Alkanes were proven to be present at peak 1456–1375 cm^–1^ because of CH_2_, CH_3_, and C–H bending vibrations, while peaks at 1231 cm^–1^ displayed C–H stretching vibrations, indicating alkanes.^51^ As a result of the C–O–C stretching vibration, the IR band at 1111–1023 cm^–1^ verified the existence of ester functionalities. Evidence of an aromatic component in the pyrolytic oil came from the peaks at 906 and 720 cm^–1^. Hemicellulose, cellulose, and lignin, which can be employed for various purposes, were proven to be present by the availability of aliphatic and a reduced amount of aromatic compounds.^52^ From Figure 5, it was clear that raw ANH biomass had very different peaks compared to those of the thermal and catalytic pyrolysis oil. However, the FTIR curve obtained from thermal and catalytic pyrolysis shows slightly different peaks at 1375, 1231, 1111, and 600 cm^–1^, respectively, due to variations in the composition of the pyrolysis oil. Further, it was also noted that the catalytic FTIR curve has a higher depth peak over thermal pyrolytic oil, confirming the improved properties of pyrolysis oil.

### 3.7 GC-MS Analysis

**Figure 6:**
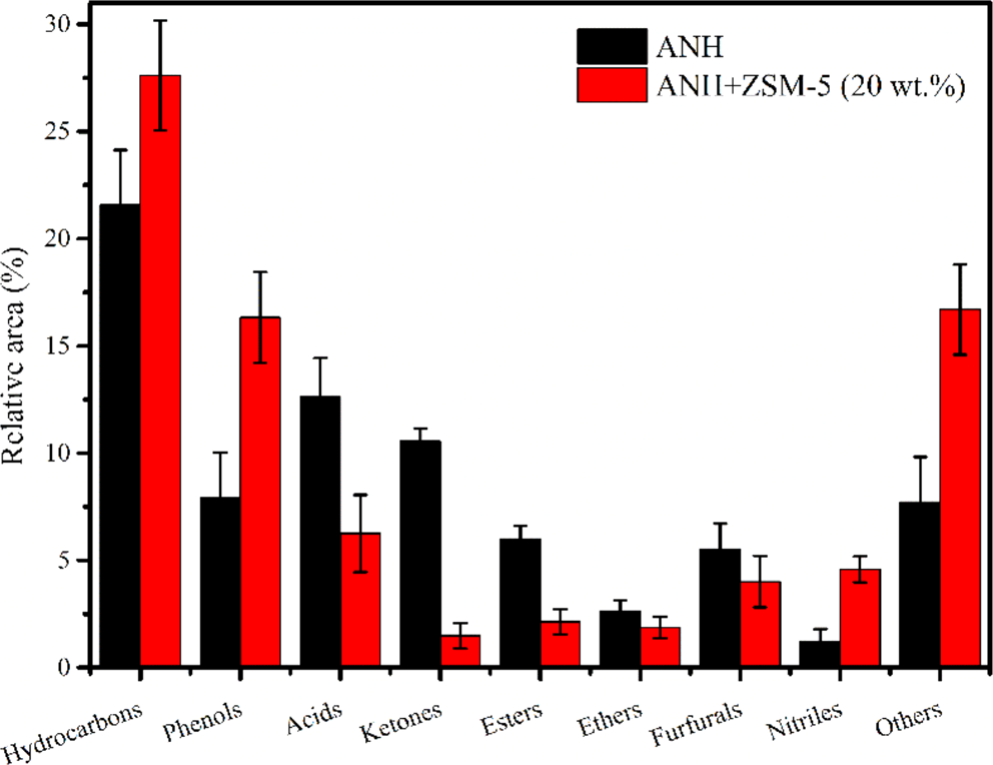
Compositional study of bio-oil derived from the catalytic pyrolysis of ANH.

**Figure 7:**
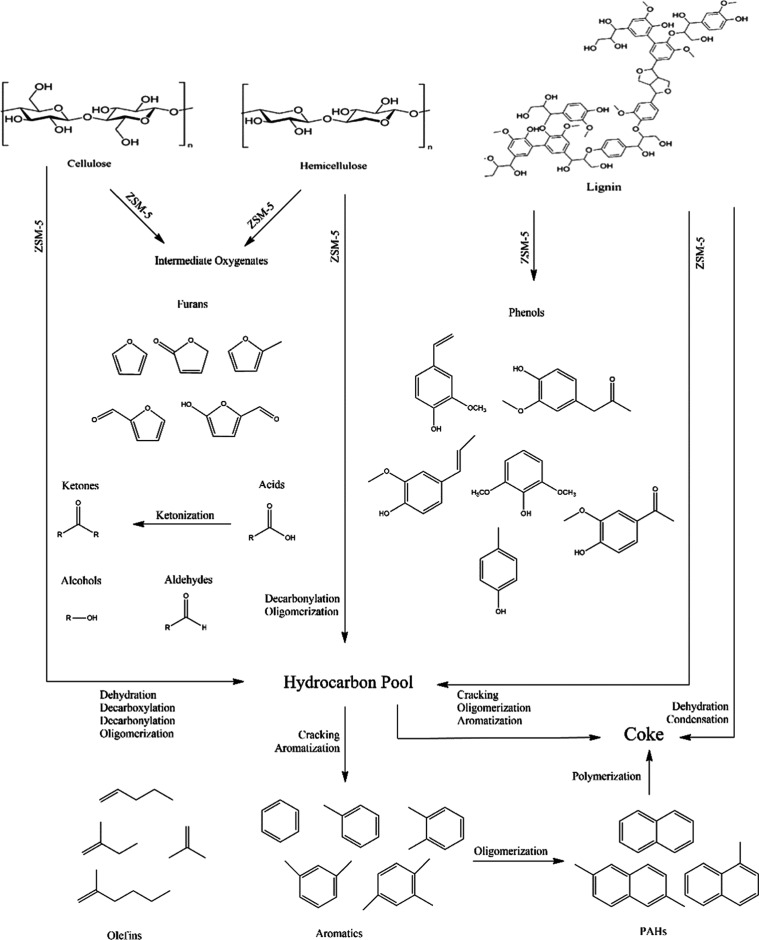
Reaction pathway for catalytic pyrolysis of ANH over ZSM-5.

The chromatograph produced by GC-MS analysis of pyrolytic oil after thermal and catalytic pyrolysis was analyzed in the NIST database and shown in Figure 6 and Tables S1 and S2 (Supporting Information). Pyrolytic oil is appropriate for use in engines since it contains a significant amount of hydrocarbons as well as aromatics, acids, esters, phenols, furan derivatives, alkanes, ketones, ethers, and aldehydes. More curiously, it may not be possible to predict the precise chemical reactions of the pyrolytic oil because the chemical components of the biomass utilized to produce pyrolytic oil may fluctuate.^23,53^ Hemicellulose and cellulose break down into acids, ketones, cycloalkanes, furanic compounds, and mixed hydrocarbons as the primary byproducts. In contrast, the majority of the hydrocarbons produced by the breakdown of lignin include guaiacyl, p-hydroxyphenyl, syringyl, aromatic hydrocarbons, and other hydrocarbons.^54^ The results showed that 21.57% of hydrocarbons, 7.91% of phenols, 12.63% of acids, 10.55% of ketones, 6% of esters, 2.64% of ethers, 5.51% of furfural, 1.2% of nitriles, and 7.72 others are processed by thermal pyrolysis oil. Similar results were also reported by pyrolysis of sal sawdust using CaO, CuO, and Al_2_O_3_.^23^ Acids would be viewed unfavorably when the primary purpose is utilizing pyrolytic oil as a transportation fuel because they adversely influence the fuel’s characteristics.^55^ 7-Tetradecene, benzene, propyl, 6-dodecene, 5-tetradecene, tricosene, tetratetracontane, benzene, etc. are the major hydrocarbon peaks which are supported by the FTIR results. Further, phenol, 2-methyl, benzene, *n*-butyl, p-Cresol, phenol, 3,5-dimethyl, phenol, phenol, 3-phenoxy, etc., are the major phenolic compounds in the pyrolysis oil. Benzene (1,2-dimethylpropyl), benzene, octadecane, 1-(ethenyloxy), etc., are the major ethers present in the pyrolysis oil. Esters were also found in significant amounts, such as ethyl 2-hydroxypropanoate, ethyl 1–2-hydroxybutanoate, 9-octadecenoic acid, methyl ester, etc., in the pyrolysis oil. Furthermore, furfurals, 5-hydroxymethylfurfural, ethylfurfural, and 2-furancarboxaldehyde are the major compounds present in pyrolysis oil. Acetic acid, hexadecenoic acid, nonanoic acid, n-hexadecanoic acid, and octadecadienoic acids are the acid compounds found in the pyrolysis oil. Ketones such as 2-cyclopenten-1-one, 2,3-dimethyl, cyclohexanone, 2-ethyl, cyclohexene, and 1-phenyl compounds are also found in the pyrolysis oil, which is supported by FTIR examination. Thermal pyrolysis oil has lower hydrocarbons (21.57%) than catalytic pyrolysis oil (27.60%). ZSM-5 catalysts in biomass pyrolysis increase the production of hydrocarbons due to a number of important features. The production of hydrocarbon intermediates is first made easier by ZSM-5′s potent acidity and shape selectivity, which encourage the breaking down of bigger biomass molecules into smaller pieces. ZSM-5 also facilitates deoxygenation processes, which remove oxygen functional groups from biomass molecules and promote the synthesis of hydrocarbons. Furthermore, ZSM-5 exhibits the ability to selectively catalyze processes that lead to the formation of hydrocarbons while minimizing the generation of other byproducts, thanks to its distinct pore structure. In catalytic pyrolytic oils, the content of acidic products experienced a significant decrease (6.11%) compared to that in thermal pyrolytic oil, primarily attributed to the conversion of acids into ketones and alcohols. In addition, the catalytic pyrolytic oil had a higher concentration of phenols (8.41%) than the thermal pyrolytic oil. ZSM-5 catalysts improve the synthesis of phenolic ingredients through a variety of processes, which makes them essential for biomass pyrolysis. Initially, the synthesis of phenolic intermediates is aided by their high acidity, which also helps dehydrate and split oxygen-containing biomass molecules. Further, shape-selective catalysis is made possible by ZSM-5′s distinct pore structure, which promotes the conversion of larger biomass molecules into smaller aromatic substances like phenols. ZSM-5 plays a crucial role in facilitating hydrogen transfer, catalytic cracking, and deoxygenation reactions during pyrolysis. These mechanisms collectively contribute to the production of phenolic chemicals. ZSM-5 catalysts are useful instruments in the processes of converting biomass as they exploit these catalytic characteristics to considerably enhance the yield of phenols in the bio-oil that is prepared from biomass.^56^ Catalysts dramatically boosted the moisture contents of the pyrolytic oil by combining oxygen and hydrogen units to form a reaction, decreasing the quantity of esters and ethers, and promoting the reformation of acids into aldehydes.^57^ By altering the deamination reaction, the catalysts that were used also diminished the nitrogen-containing products (this is due to the inhibition of dehydration of amides, which produces nitriles).^58^ The disintegration of hemicellulose and celluloses would primarily produce such chemicals, including catalysts, which increased the ratio of furans and their derivatives.^58^ Due to the accelerated dehydration reaction, the water contents of biomass disappear at a temperature of about 100 °C. Even during pyrolysis, this event can dramatically boost the quantity of amorphous carbon in the produced biochar.^7^ The reaction pathway during the pyrolysis process, including biochar production, depolymerization, and fragmentation, is typically influenced by feedstock characteristics such as elemental and proximate analysis, as well as the type of chemical bonds present, along with operating conditions. The solid waste product known as biochar, which is produced during the pyrolysis of biomass, contains an aromatic polycyclic framework.^7^ The main method for creating biochar involves the synthesis of benzene rings and their attachment to polycyclic structures. Biomass macromolecules (such cellulose and hemicellulose) break down into minute aromatic monomers and low molecular weight saturated substances at temperatures between 300 and 450 °C.^59^ Short-chain molecules and volatile substances that can condense at room temperature could arise as a result of this breakdown.^59^ Covalent bonds inside monomer units are linked during fragmentation, which produces noncondensable gases and straight short-chain products.^60^ More processes, including cracking and repolymerization, might be applied to the volatile matter to produce components with higher molecular weights. Large molecular weight materials are not typically volatile at pyrolysis temperatures, but they may be retained in the liquid or solid phase products. The biomass is decomposed into a variety of compounds against the temperature and catalysts. Figure 7 shows the reaction pathway of the decomposition of ANH over ZSM-5 at 600 °C. The biomass constituents such as hemicellulose, cellulose, and lignin decomposed in various steps and provided many compounds. The hemicellulose and cellulose pass through initially intermediate oxygenates and are further converted into furans. The furans further decompose into acids, and acids transform into ketone groups. After undergoing a decarboxylation reaction, it resulted in the formation of aromatic, olefinic, and polyaromatic compounds. Additionally, light-oxygenated compounds remove CO and CO_2_, generating hydrocarbons. Lignin was depolymerized into aromatic compounds using cracking and dehydration reactions, resulting in aromatic compounds along with other radicals. The olefinic compounds undergo further reactions, resulting in the formation of a variety of hydrocarbons and coke during the pyrolysis of biomass over ZSM-5.

## 4 Conclusions

The current study deals with the thermocatalytic and kinetic study of ANH using a semibatch reactor at optimized conditions (600 °C, 80 °C min^–1^ heating rate, and 100 mL min^–1^ nitrogen flow rate). The kinetic study of ANH confirmed that activation energy depends on the conversion value (0.1–0.8) and temperature of the pyrolysis. Further, the pyrolysis test confirmed that the introduction of a catalyst under optimized conditions significantly boosted the pyrolytic oil properties. An FTIR study of pyrolytic oil revealed the presence of OH, C–H, C=O, etc., functional groups, which established the existence of phenols, ethers, esters, alcohols, acids, ketones, alkanes, etc. Finally, the GC-MS study of pyrolytic oil demonstrated an improved percentage of hydrocarbons and phenols and a reduction in acids and other oxygenated compounds. Overall, the present study confirmed the bioenergy potential of ANH to be exploited as a renewable fuel source and chemical feedstock.

## Data Availability

The data sets generated during and/or analyzed during the current study are available from the corresponding author upon reasonable request.

## Supporting Information Available

Tables S1 and S2 contain the details of the compound analysis of bio-oil using GC-MS (PDF)
