# Supplementary material for: Thermocatalytic Pyrolysis of Waste Areca Nut into Renewable Fuel and Value-Added Chemicals
Source: ACS Omega. 2024 Jun 10;9(24):25779–92. doi: 10.1021/acsomega.3c10184 (PMC11190932; doi:10.1021/acsomega.3c10184)
Supplement: Supplementary file 1 — ao3c10184_si_001.pdf [file ao3c10184_si_001.pdf]

Thermocatalytic pyrolysis of waste areca nut into renewable fuel and value-added chemicals

Ranjeet Kumar Mishra[1]*, Bhavana Gariya[2], Priyanka Savvasere[2], Devanshu Dhir[2], Pradeep Kumar[3] Kaustubha Mohanty[4]

[1*]Department of Chemical Engineering, Manipal Institute of Technology, Manipal Academy of Higher Education, Manipal, Karnataka 576104, India

[2]Department of Chemical Engineering, Ramaiah Institute of Technology, Bangalore, Karnataka, India-560054

[5]Department of Chemical Engineering and Technology, Indian Institute of Technology (BHU), Varanasi-221005, India

[4]Department of Chemical Engineering, Indian Institute of Technology Guwahati, Assam India-781039

*Author to whom correspondent should be addressed.

Electronic Mail. ranjeet.mishra@manipal.edu (R. K. Mishra)

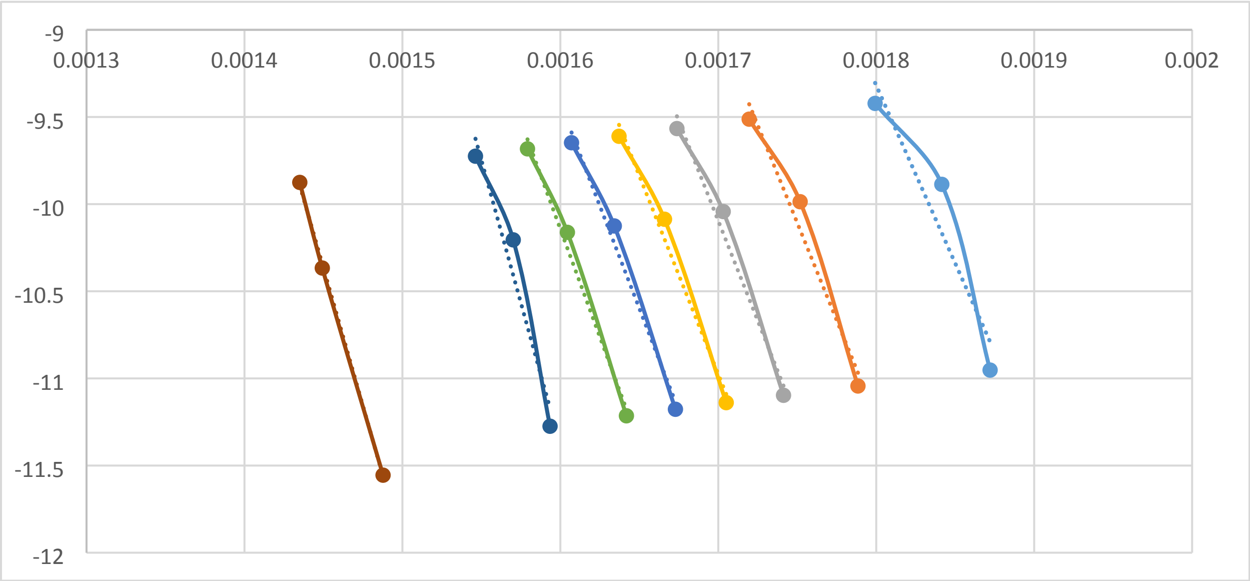

Fig. S1. Kinetics plot of ANH using KAS model.

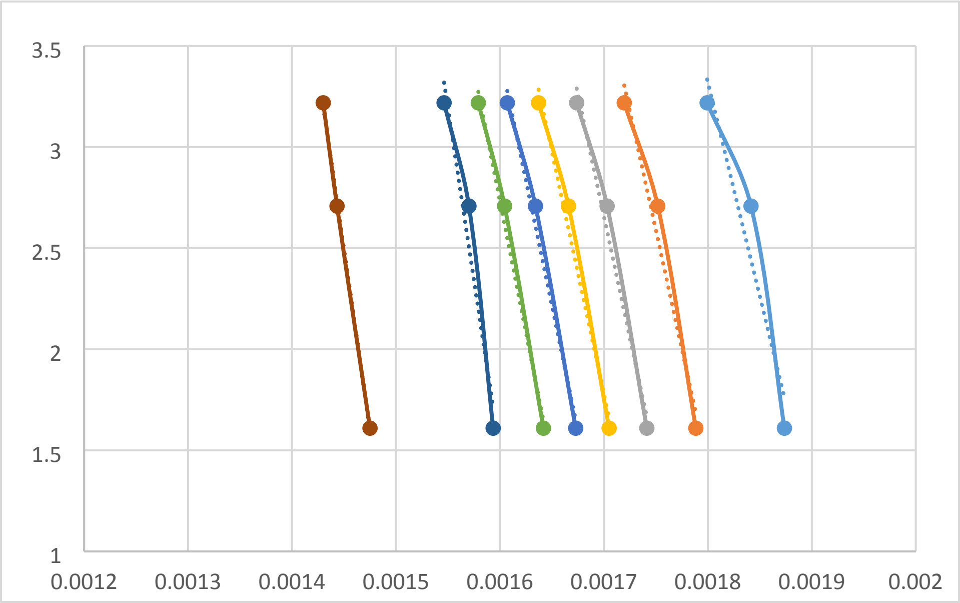

Fig. S2. Kinetics plot of ANH using OFW model.

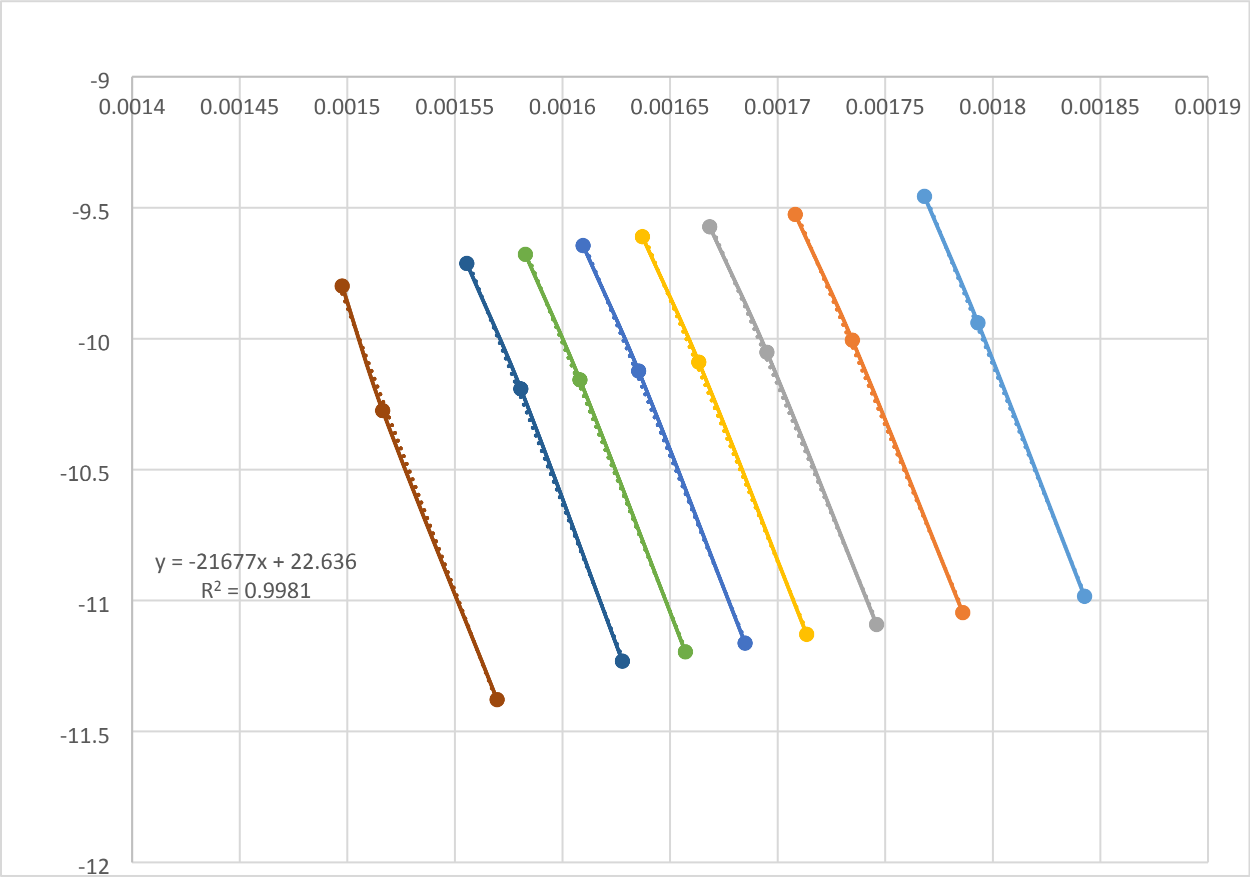

Fig. S3. Kinetics plot of ANH using DAEM model.

Table S1. Compositional analysis of pyrolysis oil obtained from pyrolysis of ANH.

R.T	Molecular weight	Compounds name	Relative area (%)
		**Hydrocarbons**	
8.05	120	Benzene, propyl	1.15
13.63	128	Naphthalene	0.63
15.16	196	7-Tetradecene	1.13
17.77	168	6-Dodecene, (E)-	1.05
17.97	506	Hexatriacontane	1.25
20.49	196	3-Tetradecene, (E)-	0.79
22.86	222	8-Hexadecyne	0.78
23.24	196	5-Tetradecene, (E)-	2.68
23.41	268	Nonadecane	0.62
24.81	246	Benzene, (1-propylnonyl)-	0.58
25.58	322	9-Tricosene, (Z)-	1.82
26.11	618	Tetratetracontane	1.2
26.36	232	Benzene, (1-methyldecyl)-	0.62
27.73	194	7-Tetradecyne	0.25
31.8	148	Benzene, (1,2-dimethylpropyl)-	0.95
10.06	134	Benzene, n-butyl	2.11
12.25	148	Benzene, pentyl	1.26
12.33	235	Benzene, (1,2-dimethylpropyl)-	1.62
12.65	240	Benzene, (1,2-dimethylpropyl)-	0.74
16.23	224	Naphthalene, 1,4-dimethyl	0.34
			**21.57**
		**Phenols**	
9.95	108	Phenol, 2-methyl	2.2
10.38	108	p-Cresol	2.24
12	122	Phenol, 3,5-dimethyl	1.67
27.38	186	Phenol, 3-phenoxy	1.8
			**7.91**
		**Ethers**	
15.33	240	Oxirane, tetradecyl	0.53
12.98	241	Octadecane, 1-(ethenyloxy)-	0.92

10.25	160	Benzene, 1,1'-(oxydiethylidene)bis	1.19
			**2.64**
		**Esters**	
11	118	Ethyl 2-hydroxypropanoate	1.2
16	118	Ethyl 1-2-hydroxybutanoate	1.3
19.67	294	9,12-Octadecadienoic acid, methyl ester, (E,E)-	0.68
20	118	Ethyl 1-3,2-hydroxypentanoate	0.89
36.2	296	9-Octadecenoic acid, methyl ester, (E)-	1.93
			**6**
		**Furfurals**	
8.11	96	Furfural	1. 50
27	126	5-hydroxymethylfurfural	1.2
45		2-Ethylfurfural	1.4
15.67	84	2(5H)-Furanone	1.3
13.28	98	2-Furanmethanol	1.26
12.34	96	2-Furancarboxaldehyde	0.35
			**5.51**
		**Acid**	
5.23	60	Acetic acid	2.53
12.71	254	Hexadecenoic acid, Z-11-	1.96
14.79	158	Nonanoic acid	1.42
20.13	280	9,12-Octadecadienoic acid (Z,Z)-	0.82
25.29	338	Erucic acid	0.89
25.41	280	9,12-Octadecadienoic acid (Z,Z)-E	1.25
33.42	256	n-Hexadecanoic acid	3.76
			**12.63**
		**Ketones**	
9.75	110	2-Cyclopenten-1-one, 2,3-dimethyl	2.55
10.53	126	Cyclohexanone, 2-ethyl	1.54
13.94	158	Cyclohexene, 1-phenyl	2.21
14	116	4-Hydroxy-4-methylpentan-2-one	1.2

12.81	198	2-Tridecanone	0.75
24	126	2-Hydroxy-3,4-dimethylcyclopent-2-enone	2.3
			**10.55**
		**Nitrile**	
31.51	251	Heptadecanenitrile	**1.2**
		**Others**	
11.45	155	4-Piperidinone, 2,2,6,6-tetramethyl	0.81
15.97	117	Indole	1.55
18.45	131	Indole, 3-methyl	3.33
8.65	154	1-Octyn-3-ol, 4-ethyl	1.3
16.74	168	(E)-4,5-epoxy-(E)-2-decenal	0.32
16.89	168	4,5-Epoxy-2-heptenal	0.41
			**7.72**

Table S2. Compositional analysis of pyrolysis oil obtained from pyrolysis of ANH+ZSM-5 (20. wt.%).

R.T	Molecular weight	Compounds name	Relative area (%)
		**Hydrocarbons**	
9.78	118	Indane	1.2
11.27	166	5,7-Dodecadiene, (E,Z)-	1.27
12.71	168	6-Dodecene, (E)-	2.03
13.37	128	Naphthalene	1.87
13.58	166	5,7-Dodecadiene, (E,Z)-	1.3
15.17	196	7-Tetradecene	1.22
16.14	142	Naphthalene, 1-methyl	1.01
16.59	142	1H-Indene, 1-ethylidene	0.58
17.78	168	6-Dodecene, (E)-	2.15
17.97	506	Hexatriacontane	0.58
18.72	156	Naphthalene, 1-ethyl	0.78
19.06	156	Naphthalene, 2,3-dimethyl	1.2
19.66	222	8-Hexadecyne	1.32
20.49	182	6-Tridecene, (Z)-	2.21
20.67	450	Dotriacontane	1.94
22.97	222	1-Hexadecyne	1.5
23.39	212	Dodecane, 2,6,10-trimethyl-	0.72

23.71	218	Benzene, (1-methylnonyl)-	0.89
24.8	246	Benzene, (1-propylnonyl)-	1.01
25.93	168	3-Dodecene, (Z)-	0.68
26.35	232	Benzene, (1-methyldecyl)-	0.12
29.18	246	Benzene, (1-methylundecyl)-	0.62
10.06	134	Benzene, n-butyl	1.04
31.8	148	Benzene, (1,2-dimethylpropyl)-	0.36
			**27.6**
		**Phenols**	
10.37	108	p-Cresol	1.31
10.38	108	p-Cresol	2.15
13	122	2,3-Xylenol	1.3
17	124	Guaiacol	1.7
20	154	3,5-Dimethoxyphenol	1.7
36	138	4-Ethoxyphenol	2.1
36	150	5-Isopropyl-2-cresol	1.5
41	154	Syringol, c	3.6
44	154	Syringol	0.96
			**16.32**
		**Furfurals**	
8.11	96	Furfural	1. 50
27	126	5-hydroxymethylfurfural	1.26
12.34	96	2-Furancarboxaldehyde	1.32
45	82.1	2-Ethylfurfural	1.43
			**4.01**
		**Esters**	
4		Ethyl acetate	1.2
37.63	356	9-Octadecenoic acid (Z)-, 2,3-dihydroxypropyl ester	0.67
			**1.87**
		**Ethers**	
2.2	74.12	Ethyl ether	**1.1**
15.33	240	Oxirane, tetradecyl	**1.03**
			**2.13**
		**Acid**	
5.23	60	Acetic acid	1.06
8.49	116	Hexanoic acid	1.89
12.25	144	Octanoic acid	0.47

17.21	172	n-Decanoic acid	0.73
25.57	338	Erucic acid	0.75
33.44	256	n-Hexadecanoic acid	1.35
			**6.25**
		**Nitrile group**	
31.52	237	Hexadecanenitrile	1.89
38.48	227	Tetradecanamide	1.93
36.43	265	Octadecanenitrile	0.76
			**4.58**
		**Ketones**	
9	110	2-Cyclopenten-1-one, 2,3-dimethyl	0.25
9.89	126	Cyclohexanone, 2-ethyl	0.54
25.06	252	Oxacycloheptadec-8-en-2-one, (8Z)-	0.69
			**1.48**
		**Others**	
11.49	155	4-Piperidinone, 2,2,6,6-tetramethyl	1.71
13.28	98	2-Furanmethanol	1.26
15.97	117	Indole	1.5
18.45	131	Indole, 3-methyl	1.62
18.52	160	Tryptamine	0.36
35.65	162	Levoglucosan	4.58
31.5	122	4-Hydroxy-benzaldehyde	0.78
14	126.15	2-Hydroxy-3,4-dimethylcyclopent-2-enone	0.72
14.2	116.16	4-Hydroxy-4-methylpentan-2-one	1.2
42	138	Furyl-2-butanone	1.5
42.21	281	9-Octadecenamide, (Z)-	1.46
			**16.69**
